# Longitudinal relationships between changes in body composition and changes in selected metabolic risk factors (abdominal obesity and blood pressure) among South African adolescents

**DOI:** 10.7717/peerj.9331

**Published:** 2020-06-23

**Authors:** Vincent Masocha, Makama Andries Monyeki, Stanisław H. Czyż

**Affiliations:** 1 Physical Activity, Sport, and Recreation Research Focus Group, North-West University, Potchefstroom, South Africa; 2 Faculty of Physical Education, University School of Physical Education in Wrocław, Wrocław, Poland; 3 Faculty of Sport Studies, Masaryk University, Brno, Czech Republic

**Keywords:** Abdominal obesity, Hypertension, Body mass index, Longitudinal study, Adolescent

## Abstract

**Background:**

Incidence of childhood high blood pressure (BP) is increasing worldwide. This study examined the longitudinal relationship between changes in body composition (i.e. body mass index (BMI), waist circumference (WC) and percentage body fat) and selected metabolic risk factors (abdominal obesity and BP) among adolescents from the Tlokwe municipality in the North West Province of South Africa.

**Method:**

One hundred and eight-six adolescents (81 boys and 105 girls) aged 14 to 16 years participated in the study. Body composition was measured following the International Society of the Advancement of Kinanthropometry standard procedures. BMI, abdominal obesity using WC measurement, and resting BP were determined. Analysis of variance (ANOVA) for repeated measures was calculated to determine changes in anthropometric measures and body composition as well as changes in BP. Additionally, Univariate analysis of variance with repeated measures and participants as a random sample was applied. Diastolic BP (DBP) and systolic BP (SBP) were used as dependent variables and sex, age, BMI, WC, and waist-to-height ratio as independent variables.

**Results:**

Significant changes were found for stature, BMI, body mass, WC, SBP, and DBP. BMI for the total group was significant and positively related to abdominal obesity in 2012 (*r* = 0.55; *p* < 0.01) and in 2013 (*r* = 0.77; *p* < 0.01) and to SBP (*r* = 0.26; *p* < 0.05) in 2012 and (*r* = 0.17; *p* = 0.43) in 2013. BMI among the boys was significantly and positively related to abdominal obesity in 2012 (*r* = 0.83; *p* < 0.01) and 2013 (*r* = 0.91; *p* < 0.01). For the girls, BMI was significantly and positively related to abdominal obesity (*r* = 0.49; *p* < 0.01) and to SBP (*r* = 0.32; *p* = 0.05) in 2012. Boys with a higher WC in 2012 had significantly increased DBP (*p* < 0.05). Boys measured in 2012 with greater WC and BMI show a significant increase in SBP.

**Conclusions:**

BMI was positively related to BP and abdominal obesity over time. Relatively high BMI and abdominal obesity significantly increased the likelihood of elevated BP over time, especially in boys. BMI was a predictor of abdominal obesity in boys, while in girls, BMI was a predictor of both abdominal obesity and SBP. In view of the future health implications of both abdominal obesity and elevated BP, urgent strategic interventions programs aimed at increasing physical activity and advocating for well-balanced dietary practices as well as importance of keeping normal blood pressure among South African adolescents are needed.

## Introduction

Globally, both obesity and hypertension are reported to be prevalent in childhood and adolescence. Worldwide obesity prevalence was noted to have risen by an estimated 47.1% between 1980 and 2013 among children and adolescents (Ng et al., 2014), while the prevalence of prehypertension increased from 7.7% to 10% in children aged 8–17 years (Din-Dzietham et al., 2007). The rapid increase in the prevalence of both obesity and hypertension is most noticeable among low- and middle-income countries that are undergoing economic transition and whose health delivery systems are weak, such as countries in Africa and Asia (World Health Organization, 2009).

The Medical Research Council of South Africa revealed that over 17% of South African children aged between 1 and 9 years living in urban areas are obese or overweight. The first South African National Health and Nutrition Examination Survey (NHANES–1) documented a combined overweight and obesity prevalence of 13.5% for South African children aged 6–14 years (Shisana et al., 2013). This is higher than the global prevalence of 10% in school children (Gupta et al., 2012) but lower than the reported levels in the USA (18% for obesity and 32.6% for combined overweight and obesity) for children aged 6–11 years (2009–2010) (Flegal et al., 2012). According to the 2008 National Income Dynamics Study in South Africa (Bradshaw et al., 2011), 11% of girls aged 15–24 (compared with 4% in the 1998 South Africa Demographic and Health Survey, SADHS) and 20% of boys aged 25–34 (compared with 10% in the 1998 SADHS) had hypertension. In girls, 12% of 15–24-year-olds (compared with 7% in the 1998 SADHS) and 24% of 25–34-year-olds (compared with 15% in the 1998 SADHS) had hypertension. One study reported prevalence rates ranging from 9.2% to 16.4% for prehypertension and 8.4% to 24.4% for hypertension among children and adolescents residing in urban areas (Kagura et al., 2015).

Studies have reported a relationship between body mass index (BMI), body size and elevated blood pressure (BP) in children and adolescents (Al-Sendi et al., 2003; Brion et al., 2007). The relationship was shown to be positive and stronger mostly among obese children (Brion et al., 2007; Larsson, Hernell & Lind, 2011; Wang et al., 2015; Dwivedi et al., 2016). Longitudinal studies have demonstrated that children with higher BMI and waist circumference (WC) changed from initially normal (baseline values) to higher BP values at follow-up (Din-Dzietham et al., 2007; Brion et al., 2007). In a systematic review of 51 studies (Noubiap et al., 2017), it was reported that an increase in BMI was largely associated with the prevalence of elevated BP, being six times higher in obese children and adolescents than those of normal weight.

Total body obesity and central obesity (also known as abdominal obesity) are associated with an increased risk of noncommunicable diseases (NCDs) such as cardiovascular diseases (CVDs) and some forms of cancer (Després et al., 2008). Arguably,  Shen et al. (2006) indicated that the metabolic risk associated with obesity is closely correlated with a central rather than peripheral fat pattern.

In spite of this, there is a paucity of information on the longitudinal relationship between changes in body composition and metabolic risk factors in South African adolescents. The purpose of this study, therefore, was to determine the longitudinal relationship between changes in body composition and selected metabolic risk factors (abdominal obesity and BP) among adolescents from the Tlokwe municipality in the North West Province of South Africa.

## Materials & Methods

This study was part of the Physical Activity and Health Longitudinal Study (PAHLS, 2010–2014), the main objective of which was to evaluate physical activity (PA) and determine health risk factors among 14-year-old high school adolescents in Tlokwe municipality in the North West Province, South Africa, over a period of 5 years (Monyeki et al., 2012). For the purpose of this study, longitudinal data collected from 2011 to 2013 were used to determine the longitudinal relationship between changes in body composition and selected metabolic risk factors (abdominal obesity and BP) among adolescents.

### Participants

One hundred-and-eighty-six adolescents (81 boys and 105 girls) from six out of eight high schools who were part of the PAHL study participated in this study. The main aim of PAHLS, a multiple longitudinal study begun in 2010, was to collect a range of health determinants on physical and health exposure variables among 14-year-old adolescents over a period of 5 years (2010–2014). Of the six high schools, two were from the central business district and comprised mostly adolescents from families of high socio-economic status, and four schools were from the township areas and comprised adolescents from families of low socio-economic status. More detailed information about the PAHLS has been provided in previous publications (Monyeki et al., 2012). The mean age of the selected learners was 14.9 ± 0.76 years at baseline measurement in 2011, 15.6 ± 0.77 years in 2012 and 16.4 ± 0.78 years in 2013. School records and participants’ birth clinic cards were used to establish the age of the participants. In line with the 20% dropout rate from the PAHL study projected in 2010, dropout rates of 21% (2012) and 33% (2013) from the 2011 measurement point were observed. The observed dropouts were due to participant absenteeism on the days of measurement, dropout from the school or transfer from one school to another. These reasons for dropout were beyond the study’s control, hence subject attrition did not have a significant effect in the analyses of the objectives of the current study. Only healthy children whose parents gave signed consent were allowed to participate. Permission was granted by the District Manager of the Department of Basic Education in Potchefstroom, North West Province before the commencement of the data collection process. Written informed consent was obtained from the school authorities, and the parents and learners of the participating schools. The Human Research Ethics Committee (HREC) of the North-West University gave ethics clearance (Ethics number: NWU-0058-01-A1).

### Procedure

Stature, body mass, skinfold thickness (triceps and subscapular skinfolds), WC and hip circumference were measured using the International Society of the Advancement of Kinanthropometry (ISAK) standard procedures (Marfell-Jones et al., 2006). Waist-to-height ratio (WHtR) was calculated as waist divided by stature (waist/stature [cm]). BMI was calculated as body mass divided by stature squared (kg/m^2^). Subsequently, the age-specific BMI for the children was used to determine the following categories: overweight (BMI of 25 and above), normal weight (BMI between 17.5 and 24.9) and underweight (BMI less than 17.5) (Cole et al., 2007). Percentage body fat (%BF) was calculated from subscapular and triceps skinfold (mm) measurements using the equation of Slaughter et al. (1988), which has been internationally recommended for use in children from different settings. Anthropometric sites were measured twice according to standard procedures, by Level 2 ISAK-certified anthropometrists.

Abdominal obesity was determined using WC measurement. WC was measured at the abdomen at its narrowest point between the lower costal (10th rib) border and the top of the iliac crest, perpendicular to the long axis of the trunk, with a Lufkin^®^ W606PM flexible steel tape (Creative Health Products, MI, USA) according to ISAK (Marfell-Jones et al., 2006). Age-, sex-, and ethnicity-specific WC values that fall on the 75th and 90th percentile are important in the identification of children and adolescents at risk for various comorbidities such as CVDs, hyperinsulinemia and type 2 diabetes mellitus (Fernández et al., 2004).

BP measurements were taken on the left arm using an Omron MIT Elite Plus digital sphygmomanometer (Omron^®^ Healthcare Co., Ltd, Japan). Participants were asked to lie down and rest for 5 min before BP was measured; talking was not permitted during the rest period, nor when BP was being measured. The average of two separate measurements at least 5 min apart were used in the analysis. A measurement of systolic blood pressure (SBP) >130 millimetre of mercury (mmHg) and diastolic blood pressure (DBP) >85 mmHg was classified as abnormal according to the International Diabetes Federation cut point, and SBP ≥90th percentile for the whole population was considered abnormal according to the National Cholesterol Education Program/Adult Treatment Panel III (NCEP/ATP III) criteria (Jolliffe & Janssen, 2007).

### Statistical analysis

The frequency of percentages for categorical variables was calculated. Analysis of variance (ANOVA) for repeated measures was calculated to determine changes in anthropometric measures and body composition as well as changes in BP. A paired-sample *t*-test was used to determine mean change at the measurement points. Effect size (partial eta-squared [*η*^2^]) was used to determine the magnitude of change at three measurement points (test one, 2011 [T1], test two, 2012 [T2] and test three, 2013 [T3]). Greenhouse-Geisser method, with which an adjustment to the degree of freedom of the repeated-measures ANOVA is made, was used if the correlation was <0.75 and Huynh-Feldt method was used when a correlation was >0.75. Huynh-Feldt method was used for WHtR, SBP and DBP. Post-hoc analyses of Bonferroni were used to determine the time effect within subjects. Partial *η*^2^ used with ANOVA is in agreement with Cohen’s rule of thumb whereby values of partial *η*^2^ are interpreted as follows: 0.2, 0.5 and 0.8 were regarded as small, medium and large effects, respectively. Partial correlation coefficients, adjusted for initial measurement values and age, were calculated to determine the 2-year longitudinal relationship between change in body composition and change in selected metabolic risk factors (i.e., abdominal obesity and BP).

Additionally, we applied a univariate ANOVA, which considers the repeated measures, i.e., that the same participants were measured on three occasions, and regards the participants as a random sample. We performed two analyses. In the first one, DBP and in the second SBP were used as dependent variables. In both analyses, the independent variables were sex, age, BMI, WC, and WHtR. Partial *η*^2^ was used as a measure of effect size.

All data were analyzed using the Statistical Package for Social Sciences (SPSS^®^) version 25.0 (IBM SPSS^®^ Statistics v.25, Chicago, IL, USA) and the level of significance was set at *p* ≤ 0.05.

## Results

For the total group, developmental growth in terms of body stature and body mass showed a moderate effect size, with significant differences between boys and girls (Table 1). The effect size for WHtR, SBP, and DBP was small. Mean changes in stature from the first measurement (T1) to second measurement (T2) for the total group was 2.16 cm, and 0.76 cm from T2 to T3. Changes in stature at T2 and T3 in boys showed a significant (*p* < 0.001) increase of 3.75 cm and 2.7 cm respectively, compared with the girls (0.62 cm and one cm). Body mass increased by 3.61 kg from T1 to T2, and 2.54 kg from T2 to T3 for the total group; in boys the increase was significantly higher (*p* < 0.001) at 4.84 kg from T1 to T2 compared with girls at 2.42 kg from T1 to T2, and 3.26 kg from T2 to T3 compared with girls at 1.86 kg from T2 to T3. With regard to changes in BMI for the total group, increases of 0.82 kg from T1 to T2 and 0.48 kg from T2 to T3 were observed. Changes in BMI at the T2 and T3 measurement points in boys showed a significant (*p* = 0.01) increase of 0.83 kg (compared with 0.81 kg for girls) and 0.48 kg (compared with 0.46 for girls). Body composition increases over time were moderate.

**Table 1:** Participant characteristics (mean ± standard deviation) for each testing point for the total group and by sex.

	**T1 (2011)**	**T2 (2012)**	**T3 (2013)**	***t***	***F***	***df***	***p***	partial *η*^2^
**Total (*n* = 186)**								
Stature (cm)	160.57 ± 8.68	162.53 ± 9.16	164.19 ± 9.67	219.757	108.55	1.188	<0.001	0.41
Body mass (kg)	53.82 ± 12.53	57.26 ± 12.79	59.80 ± 13.41	260.641	165.48	1.416	<0.001	0.53
BMI (kg/m^2^)	20.79 ± 4.02	21.59 ± 4.03	22.11 ± 4.23	280.012	77.712	1.514	<0.001	0.35
WC (cm)	67.89 ± 8.18	69.04 ± 8.42	70.11 ± 8.74	357.122	27.409	1.930	<0.001	0.21
WHtR	.42 ±.04	.42 ±.04	.42 ±.05	369.067	1.706	1.995	0.183	0.01
SBP (mmHg)	103.45 ± 10.51	107.80 ± 12.42	109.26 ± 9.73	298.615	16.858	1.999	<0.001	0.10
DBP (mmHg)	66.70 ± 8.35	67.70 ± 8.88	71.66 ± 8.11	300.000	20.152	2.000	<0.001	0.20
**Boys (*n* = 81)**								
Stature (cm)	164.80 ± 9.07	168.55 ± 8.34	171.25 ± 8.17	95.172	113.969	1.197	<0.001	0.58
Body mass (kg)	54.46 ± 12.03	59.30 ± 13.38	62.56 ± 14.03	104.889	128.237	1.311	<0.001	0.61
BMI (kg/m^2^)	19.86 ± 2.99	20.69 ± 3.34	21.17 ± 3.52	114.240	42.071	1.428	<0.001	0.34
WC (cm)	67.63 ± 6.87	69.81 ± 7.74	71.09 ± 8.34	119.585	42.279	1.495	<0.001	0.35
WHtR	.41 ±.03	.41 ±.03	.41 ±.04	123.771	1.547	2.051	0.144	0.02
SBP (mmHg)	106.55 ± 11.68	111.68 ± 12.52	112.15 ± 7.93	140.00	2.000	7.813	0.001	0.10
DBP (mmHg)	68.30 ± 9.24	70.23 ± 9.73	73.04 ± 8.02	115.423	1.749	2413.296	<0.001	0.93
**Girls (*n* = 105)**								
Stature (cm)	158.25 ± 7.09	158.87 ± 7.22	159.87 ± 7.36	164.900	1.586	38.567	<0.001	0.27
Body mass (kg)	54.56 ± 14.27	56.98 ± 13.56	58.84 ± 13.81	162.812	59.070	1.565	<0.001	0.36
BMI (kg/m^2^)	21.68 ± 4.99	22.49 ± 4.80	22.95 ± 4.88	159.003	38.933	1.529	<0.001	0.27
WC (cm)	68.13 ± 9.31	68.30 ± 9.01	69.18 ± 9.06	202.675	1.949	3.375	0.04	0.03
WHtR	.43 ±.05	.43 ±.05	.43 ±.05	204.677	1.968	0.498	0.606	0.005
SBP (mmHg)	100.70 ± 8.52	104.36 ± 11.33	106.70 ± 10.49	149.064	1.887	9.459	<0.001	0.11
DBP (mmHg)	65.29 ± 7.23	65.46 ± 7.42	70.44 ± 8.04	151.228	1.914	3,653.287	<0.001	0.98

**Notes.**

T1 test one T2 test two T3 test three t *t*-test within-subject factor F time effect df degree of freedom BMI Body mass index DBP diastolic blood pressure SBP systolic blood pressure SD standard deviation WC waist circumference WHtR weight-to-height ratio

Increases of 1.15 cm in WC from T1 to T2 and 1.07 cm from T2 to T3 were found for the total group, with boys showing a significant (*p* = 0.01) increase of 2.18 cm from T1 to T2 (compared with 0.17 cm for girls) and 1.28 cm from T2 to T3 (compared with 0.88 cm for girls). Mean change in SBP for the total group between T1 and T2 was 4.35 mmHg, and 1.46 mmHg from T2 to T3. Boys presented significantly (*p* < 0.001) greater mean changes at the T2 and T3 measurement points (5.13 mmHg and 2.34 mmHg, compared with 3.66 mmHg and 0.47 mmHg for girls, respectively). Mean change in DBP from T1 to T2 for the total group was one mmHg and from T2 to T3 was 3.96 mmHg. Change in DBP from T1 to T2 was significantly greater in boys (1.93 mmHg) compared with girls (1.7 mmHg), while between T2 and T3, girls presented with significantly (*p* < 0.001) greater change in DBP (4.98 mmHg) compared with boys (2.81 mmHg). The magnitude of change in BP was small over time.

The developmental tracking correlation coefficients for stature, body mass, BMI, WHtR and WC were significantly high (*p* ≤ 0.05) for the total group and for boys and girls (Table 2). For BP, SBP development showed low significance between the T1 and T2 measurement points (*r* = .39; *p* = 0.01) as well as between the T2 and T3 measurement points (*r* = .23; *p* = 0.01), for the total group. In terms of sex, a significant development was found at measurements points T1 and T2 in boys, while in girls, a significant development was found at the measurement points T2 and T3 for both SBP and DBP.

**Table 2:** Correlation coefficients of the T1 measurements with the T2 and T3 measurements.

	**Total group**		**Boys**		**Girls**	
	**T2 (2012)**	**T3 (2013)**	**T2 (2012)**	**T3 (2013)**	**T2 (2012)**	**T3 (2013)**
	r	r	r	r	r	r
Stature (cm)	.90^**^	.88^**^	.91^**^	.87^**^	.86^**^	.93^**^
Body mass (kg)	.93^**^	.90^**^	.91^**^	.82^**^	.93^**^	.94^**^
BMI (kg/m^2^)	.92^**^	.88^**^	.91^**^	.84^**^	.92^**^	.91^**^
WC (cm)	.89^**^	.87^**^	.87^**^	.80^**^	.89^**^	.90^**^
WHtR	.86^**^	.83	.76^**^	.69^**^	.90^**^	.83^**^
SBP (mmHg)	.39^**^	.23^**^	.43^**^	−.05	.21	.38^**^
DBP (mmHg)	.15	.16	.37^**^	.08	.04	.24^*^

**Notes.**

** Correlation is significant at the 0.01 level (two-tailed).

* Correlation is significant at the 0.05 level (two-tailed).

BMI Body mass index DBP diastolic blood pressure SBP systolic blood pressure WC waist circumference WHtR waist-to-height ratio

**Figure 1:**
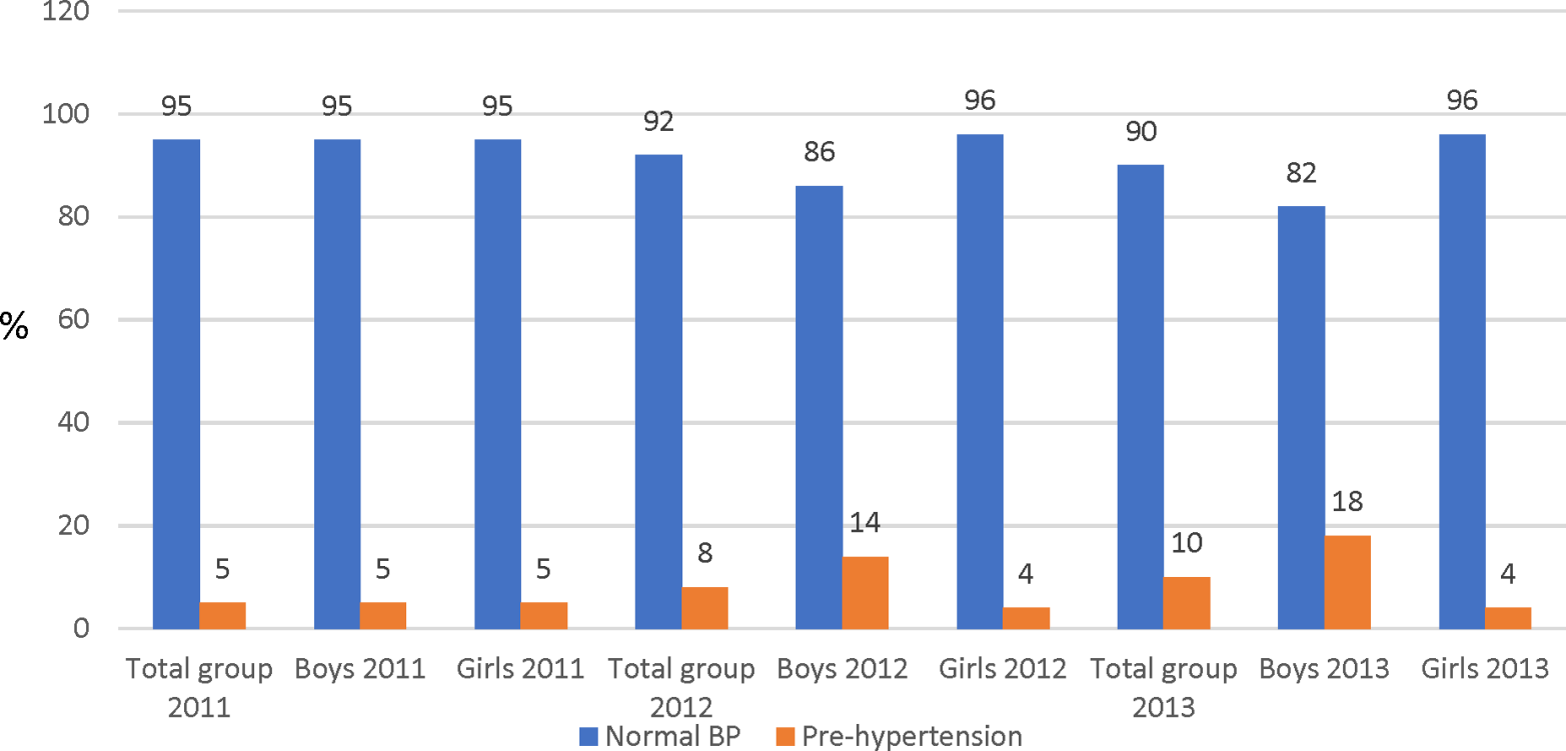
Percentage score (%) of prehypertension of the total participants and by sex.

In the total group and for boys and girls at the T1 measurement point, prevalence of prehypertension was 5.0% (Fig. 1). At the T2 measurement point, prehypertension had increased by 3.0% for the total group, and a 9.0% in boys. An increase of 2% in prevalence prehypertension from T2 to T3 was observed for the total group, with boys demonstrating a high percentage (18%) compared with the girls (4.0%). In girls, prehypertension decreased by 1% in T2 and remained unchanged at T3.

**Figure 2:**
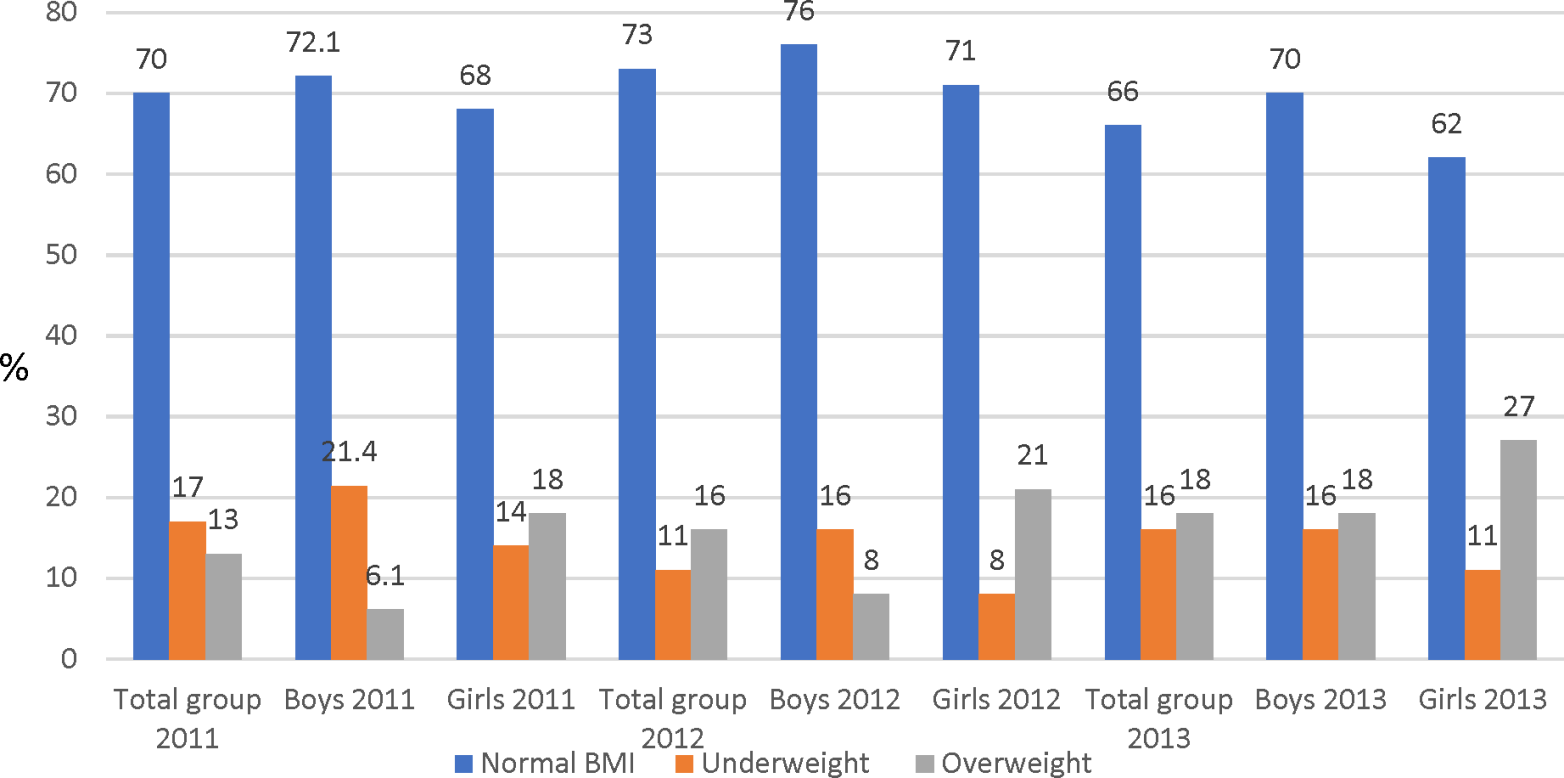
Percentage (%) scores of BMI categories distribution for the total participants and sex.

The prevalence of overweight at the first measurement point was 13.0% for the total group, with a greater proportion of girls being overweight (18.0%) than boys (6.1%) (Fig. 2). The proportion of underweight subjects was 17% for the total group with boys (21.4%) more underweight than girls (14.0%). There was an increase in the proportion of children who were overweight and a decrease in those underweight over the three measurement points, and girls were more overweight and boys more underweight over the three time points.

The correlation matrix (Table 3) showed that BMI adjusted for age and baseline measurements for the total group was significantly and positively related to WC, WHtR and SBP at T2 and T3 measurement points.

When data were analyzed separately according to sex, BMI among the boys (Table 4) was significantly and positively related to WC and WHtR (*p* < 0.01) at all measurement points. The relationship between BMI and BP among the boys was in a positive direction although it was not statistically significant. Among the girls, BMI adjusted for age and baseline measurements was significantly and positively related to WC and WHtR (*p* < 0.01) at all measurement points. A positive significant relationship was also noted between BMI and SBP at the T2 measurement point (*p* = 0.01), while at the T3 measurement point a borderline relationship was found and WHtR was significantly related to SBP (*p* = 0.05).

**Table 3:** Correlation matrix of the three point measurements of anthropometry, body composition and blood pressure for the total group.

		^Δ^**BMI T2**	^Δ^**WHtR T2**	^Δ^**BMI T3**	^Δ^**WHtR T3**	^Δ^**WC T2**	^Δ^**WC T3**	^Δ^**SBP T2**	^Δ^**DBP T2**	^Δ^**SBP T3**	^Δ^**DBP T3**
BMI T2	r	–	.59	.82	.59	.55	.52	.26	.09	.08	.04
	p	.	.00	.00	.00	.00	.00	.00	.26	.33	.65
WHtR T2	r	.59	–	.49	.64	.92	.59	.10	.15	.11	.05
	p	.00	.	.00	.00	.00	.00	.23	.07	.19	.59
BMI T3	r	.82	.49	–	.83	.46	.77	.19	.06	.17	.07
	p	.00	.00	.	.00	.00	.00	.03	.52	.04	.43
WHtR T3	r	.59	.64	.83	–	.58	.90	.05	.06	.14	.09
	p	.00	.00	.00	.	.00	.00	.52	.49	.09	.24

**Notes.**

Δ Change Adjusted for age, first measurements of BMI, WHtR, SBP and DBP *p* *p*-value of the significant change BMI Body mass index DBP diastolic blood pressure SBP systolic blood pressure WC waist circumference WHtR waist-to-height ratio

**Table 4:** Correlation matrix of the three point measurements of anthropometry, body composition and blood pressure for the boys and girls.

		Boys (*n* = 81)										Girls (*n* = 105)									
		^Δ^**BMI T2**	^Δ^**WHtR T2**	^Δ^**BMI T3**	^Δ^**WHtR T3**	^Δ^**WCT2**	^Δ^**WCT3**	^Δ^**SBPT2**	^Δ^**DBP T2**	^Δ^**SBP T3**	^Δ^**DBP T3**	^Δ^**BMI T2**	^Δ^**WHtR T2**	^Δ^**BMI T3**	^Δ^**WHtR T3**	^Δ^**WC T2**	^Δ^**WC T3**	^Δ^**SBP T2**	^Δ^**DBPT2**	^Δ^**SBP T3**	^Δ^**DBP T3**
BMI T2	r	–	.80	.84	.73	.83	.75	.11	.09	.14	.02	–	.51	.81	.52	.50	.52	.33	.14	.07	.02
	p	.	.00	.00	.00	.00	.00	.37	.44	.27	.89	–	.00	.00	.00	.00	.00	.01	.24	.57	.87
WHtR T2	r	.80	–	.70	.76	.90	.70	.09	.09	.07	.02	.51	–	.39	.57	.97	.54	.06	.18	.11	.02
	p	.00	.	.00	.00	.00	.00	.49	.46	.57	.89	.00	–	.00	.00	.00	.00	.65	.14	.39	.84
BMI T3	r	.84	.70	–	.91	.72	.91	.06	.01	.13	.04	.81	.39	–	.75	.37	.75	.19	.06	.22	.04
	p	.00	.00	.	.00	.00	.00	.61	.95	.30	.73	.00	.00	–	.00	.00	.00	.12	.65	.06	.76
WHtR T3	r	.73	.76	.91	–	.69	.91	−.01	−.02	.05	.01	.52	.57	.75	–	.56	.97	.02	.11	.23	.14
	p	.00	.00	.00	.	.00	.00	.92	.86	.70	.92	.00	.00	.00	–	.00	.00	.85	.35	.05	.24

**Notes.**

Δ Change; Adjusted for age, first measurements of BMI, WHtR, SBP and DBP *p* *p*-value of the significant change BMI Body mass index DBP diastolic blood pressure SBP systolic blood pressure WC waist circumference WHtR waist-to-height ratio

### Univariate analysis of variance

In the first univariate analysis we used DBP as a dependent variable. Variables of sex, measurement time (T1, T2, and T3), and WC were statistically significant (Table 5).

**Table 5:** Univariate analysis of variance: test of between-subjects effect with dependent variable diastolic blood pressure.

Source		*df*	Mean Square	*F*	p	partial *η*^2^
Intercept	Hypothesis	1	2,640.702	40.439	0.000	0.070
	Error	534.531	65.301^a^			
Sex	Hypothesis	1	544.143	8.544	0.004	0.014
	Error	624	63.687^b^			
Age	Hypothesis	1	53.250	0.836	0.361	0.001
	Error	624	63.687^b^			
Time	Hypothesis	2	927.805	14.568	0.000	0.045
	Error	624	63.687^b^			
BMI	Hypothesis	1	4.246	0.067	0.796	0.000
	Error	624	63.687^b^			
WC	Hypothesis	1	321.760	5.052	0.025	0.008
	Error	624	63.687^b^			
WHtR	Hypothesis	1	15.830	0.249	0.618	0.000
	Error	624	63.687^b^			

**Notes.**

a 0.002 MS (time) + .998 MS(error).

b MS(error).

Parameter estimation is presented in Table 6. Being a male with a higher WC at T3 significantly increased (*p* < 0.05) DBP. Measurement points (i.e., having been measured at T1 or T2) had the greatest effect on DBP (highest partial *η*^2^, 0.039 and 0.03, respectively for T1 and T2). The earlier the measurements were taken, the lower the DBP (see Table 6).

In the second analysis, SBP was used as a dependent variable (Table 7). Unlike in the previous analysis, all independent variables except age were significant (*p* < 0.05).

**Table 6:** Univariate analysis of variance: parameter estimates with dependent variable diastolic blood pressure.

Parameter	B	Std. Error	t	*p*	95% Confidence Interval		partial *η*^2^
					Lower Bound	Upper Bound	
Intercept	49.671	7.988	6.218	0.000	33.985	65.357	0.058
Boys	2.376	0.813	2.923	0.004	0.780	3.972	0.014
Girls	0^a^						
Age	0.414	0.452	0.914	0.361	−0.475	1.302	0.001
T1 (2011)	−4.153	0.822	−5.053	0.000	−5.767	−2.539	0.039
T2 (2012)	−3.525	0.804	−4.385	0.000	−5.104	−1.947	0.030
T3 (2013)	0^a^						
BMI	−0.049	0.191	−0.258	0.796	−0.425	0.326	0.000
WC	0.278	0.124	2.248	0.025	0.035	0.521	0.008
WHtR	−10.508	21.076	−0.499	0.618	−51.897	30.881	0.000

**Notes.**

a This parameter is set to zero because it is redundant.

BMI Body mass index WC waist circumference WHtR waist-to-height circumference

**Table 7:** Univariate analysis of variance: test of between-subjects effect with dependent variable systolic blood pressure.

Source		*df*	Mean Square	*F*	p	partial *η*^2^
Intercept	Hypothesis	1	10393.367	103.815	0.000	0.158
	Error	554.617	100.114^a^			
Sex	Hypothesis	1	2321.870	23.704	0.000	0.037
	Error	624	97.951^b^			
Age	Hypothesis	1	28.332	0.289	0.591	0.000
	Error	624	97.951^b^			
Time	Hypothesis	2	1256.344	12.826	0.000	0.039
	Error	624	97.951^b^			
BMI	Hypothesis	1	502.643	5.132	0.024	0.008
	Error	624	97.951^b^			
WC	Hypothesis	1	453.227	4.627	0.032	0.007
	Error	624	97.951^b^			
WHtR	Hypothesis	1	560.589	5.723	0.017	0.009
	Error	624	97.951^b^			

**Notes.**

a 0.002 MS (time) + .998 MS (error).

b MS (error).

BMI Body mass index WC waist circumference WHtR waist-to-height ratio

Being male measured at T3 with greater WC and BMI and lower WHtR, significantly increased SBP (Table 8).

**Table 8:** Univariate analysis of variance: parameter estimates with dependent variable of systolic blood pressure.

Parameter	B	Std. Error	t	*p*	95% Confidence Interval		partial *η*^2^
					Lower Bound	Upper Bound	
Intercept	100.120	9.906	10.107	0.000	80.667	119.573	0.141
Boys	4.908	1.008	4.869	0.000	2.928	6.887	0.037
Girls	0^a^						
Age	0.302	0.561	0.538	0.591	−0.800	1.403	0.000
T1 (2011)	−4.507	1.019	−4.422	0.000	−6.509	−2.506	0.030
T2 (2012)	−0.402	0.997	−0.403	0.687	−2.359	1.556	0.000
T3 (2013)	0^a^						
BMI	0.537	0.237	2.265	0.024	0.072	1.003	0.008
WC	0.330	0.154	2.151	0.032	0.029	0.632	0.007
WHtR	−62.530	26.138	−2.392	0.017	−113.859	−11.201	0.009

**Notes.**

a This parameter is set to zero because it is redundant.

BMI Body mass index WC waist circumference WHtR waist-to-height ratio

## Discussion

The purpose of our study was to determine the 2-year longitudinal relationship between changes in body composition and selected metabolic risk factors in 14-year-old adolescents from Potchefstroom in the North West Province of South Africa. The major findings (using univariate ANOVA for repeated measures) were that sex (boys) and T3 were significant predictors of higher SBP and DBP. In girls, changes in BMI and abdominal obesity were positively correlated with SBP. It has to be considered, however, that the effect size as measured by partial *η*^2^was relatively small. Higher SBP can be also explained by BMI, WC and WHtR.

Our findings were consistent with studies from schools in Mexico City (Flores-Huerta et al., 2009) and Indianapolis (Tu et al., 2011), and in Chinese and Brazilian children (Wang et al., 2015; Zhao et al., 2017), in which strong positive relationships between obesity and high BP were reported. Given our observed findings and the disease outcomes seen in adults with the relationships observed between BP and obesity, strategic intervention to prevent the development of cardiometabolic disease is needed.

The risk of hypertension increases as the odds ratio of overweight and obesity increases (Li et al., 2008). Our study findings showed that an increase in BMI predicts an increase in both abdominal obesity and BP. In this regard, maintaining a normal body weight could be a measure for preventing the development of hypertension.

A lack of a significant relationship between BMI and BP in boys was also reported among Chinese children (Wang et al., 2015). In our study, the non-significant relationship between BMI and BP in boys compared with girls could be explained by a small increase in BMI among the boys compared with the girls throughout the measurement period.

The prevalence of overweight for the total sample was 13%, with girls more overweight (18%) than boys (14%). The prevalence of overweight in this study was lower than that of adolescents in the Grand Canary Islands (29.1%) (Henríquez et al., 2008) and the Balearic Islands (24.7%) (Bibiloni et al., 2010), but greater than the 11.3% of adolescents in the CASPIAN study (Iran) (Kelishadi et al., 2008) and Chinese adolescents (4.4%) (Li et al., 2008). The prevalence of underweight was 17% for the total sample, with boys (21.4%) more underweight than girls (14%). This was greater than the 13.9% (total sample), 8.1% (boys) and 5.7% (girls) reported by Li et al. (2008) although there was consistency in that boys were more underweight than girls in both samples. The tendency for boys to have greater WC than girls in our study agrees with the findings of De Moraes et al. (2011), who reported a higher prevalence of abdominal obesity among boys than girls.

High developmental tracking correlation coefficients were found in our study for body mass, BMI, WHtR and WC, showing that the upward trends in obesity for South African children and adolescents are no different to other children across the world. Childhood obesity is widely reported to be on the rise globally (World Health Organization, 2009; Wabitsch, Moss & Kromeyer-Hauschild, 2014; Tremblay et al., 2016). Motorized transportation, low levels of PA and more time spent in sedentary behavior (although not included in the analyses of our current study) may be blamed for the high prevalence of overweight and obesity among the youth worldwide (Ng et al., 2014; De Moraes et al., 2011; Onywera et al., 2012). The increase in indices of adiposity found in this study also confirmed the findings from earlier studies that childhood obesity is on the rise among South African children and adolescents (Draper et al., 2014; Pienaar, 2015).

Elevated BP during childhood and adolescence is associated with increased cardiovascular risk in later life and the development of early pathological signs of atherosclerosis (Brion et al., 2007). Boys presented with high percentage prevalence of prehypertension (14% in 2012 and 18% in 2013) compared with a respectively 4.0% in 2012 and 2013 for girls. The higher prehypertension prevalence in boys could be explained by a greater increase in WC among the boys at all measurement points compared with the girls. High BMI and WC are both risk factors for high BP (Martín-Espinosa et al., 2017). If the situation remains unchecked, boys and girls in this sample could be at risk of developing hypertension in later life.

Some of the unique strengths of this study may have also generated limitations. The study used one age-birth cohort of boys and girls, and followed them over two year-measurement points. Also, the group of adolescent boys and girls were from selected schools in Potchefstroom town (high socio-economic status) and township (low socio-economic status); the results of the study may have differed if the study had used a larger sample of adolescents. Risk factors for overweight and obesity have been found to include low levels of PA, sedentary behavior and poor nutritional behavior (Bibiloni et al., 2010; Tremblay et al., 2016). In this study, the PA and nutritional behavior of the adolescents were not assessed, although these factors could have influenced the relationship between changes in body composition and selected metabolic risk factors.

## Conclusions

In conclusion, we found that being overweight with a moderate increasing magnitude over time was on the rise among adolescents living in the Tlokwe local municipality area in Potchefstroom. Both high BMI and abdominal obesity significantly increased the likelihood of high BP over time. BMI was a predictor of abdominal obesity in boys while in girls BMI was a predictor of both abdominal obesity and high BP. The rising trend in BMI, WC and SBP may put adolescents at risk of developing CVDs later in life. In view of the future health implications of both abdominal obesity and elevated BP, urgent strategic interventions programs aimed at increasing physical activity and advocating for well-balanced dietary practices as well as the importance of keeping normal blood pressure among South African adolescents are needed.

##  Supplemental Information

10.7717/peerj.9331/supp-1 Supplemental Information 1 Raw data

## Additional Information and Declarations

### Data Availability

The following information was supplied regarding data availability: The raw data are available as a Supplementary File with all variables and participants (coded).
